# Chlorogenic Acid Inhibits Human Platelet Activation and Thrombus Formation

**DOI:** 10.1371/journal.pone.0090699

**Published:** 2014-03-05

**Authors:** Eduardo Fuentes, Julio Caballero, Marcelo Alarcón, Armando Rojas, Iván Palomo

**Affiliations:** 1 Department of Clinical Biochemistry and Immunohematology, Faculty of Health Sciences, Interdisciplinary Excellence Research Program on Healthy Aging (PIEI-ES), Universidad de Talca, Talca, Chile; 2 Centro de Estudios en Alimentos Procesados (CEAP), CONICYT-Regional, Gore Maule, Talca, Chile; 3 Center for Bioinformatics and Molecular Simulations, Faculty of Engineering in Bioinformatics, Universidad de Talca, Talca, Chile; 4 Biomedical Research Laboratories, Medicine Faculty, Catholic University of Maule, Talca, Chile; King's College London School of Medicine, United Kingdom

## Abstract

**Background:**

Chlorogenic acid is a potent phenolic antioxidant. However, its effect on platelet aggregation, a critical factor in arterial thrombosis, remains unclear. Consequently, chlorogenic acid-action mechanisms in preventing platelet activation and thrombus formation were examined.

**Methods and Results:**

Chlorogenic acid in a dose-dependent manner (0.1 to 1 mmol/L) inhibited platelet secretion and aggregation induced by ADP, collagen, arachidonic acid and TRAP-6, and diminished platelet firm adhesion/aggregation and platelet-leukocyte interactions under flow conditions. At these concentrations chlorogenic acid significantly decreased platelet inflammatory mediators (sP-selectin, sCD40L, CCL5 and IL-1β) and increased intraplatelet cAMP levels/PKA activation. Interestingly, SQ22536 (an adenylate cyclase inhibitor) and ZM241385 (a potent A_2A_ receptor antagonist) attenuated the antiplatelet effect of chlorogenic acid. Chlorogenic acid is compatible to the active site of the adenosine A_2A_ receptor as revealed through molecular modeling. In addition, chlorogenic acid had a significantly lower effect on mouse bleeding time when compared to the same dose of aspirin.

**Conclusions:**

Antiplatelet and antithrombotic effects of chlorogenic acid are associated with the A_2A_ receptor/adenylate cyclase/cAMP/PKA signaling pathway.

## Introduction

Cardiovascular diseases (CVD) (i.e. acute myocardial infarction, cerebrovascular disease and peripheral arterial thrombosis) have increased significantly in recent years, accounting for 17.3 million deaths per year, a figure that is expected to increase to >23.6 million by 2030 [1], [2].

Plaque disruption initiates both platelet adhesion and aggregation on exposed vascular surfaces and activates the clotting cascade leading to the so-called atherothrombotic process [3]. Platelets are key mediators of inflammation as well as thrombosis through direct cell interaction [4], [5]. Platelet-endothelial cell interactions at lesion-prone sites may trigger an inflammatory response in vessel wall early in the development of atherosclerosis and contribute to the destabilization of advanced atherosclerotic lesions [6]. Reports in the last decade support the fact that the secretion of platelet-derived pro-inflammatory molecules (such as sCD40L, CCL5, IL-1β and sP-selectin) exacerbate the inflammatory response within the atherosclerotic plaque [7], [8]. Although antiplatelet drugs have proven to be beneficial in patients with clinical evidence of CVD, outcomes still remain poor. This is due to the fact that antiplatelet agents are associated with serious adverse effects (internal bleeding and gastrointestinal adverse effects, among others) [9] and their effectiveness in primary prevention is still a matter of debate [10]. Therefore, further study of antiplatelet treatment and the development of novel antiplatelet agents with increased efficacy and safety profiles is required.

The extensive association shown between diet and health demonstrates the power of nutrition in maintaining and improving health. This has provoked great interest in searching for novel products that may improve health and well-being [11]. Thus, there is increased interest in natural products isolated from plants to suppress platelet function [12]. Interestingly, some natural bioactive ingredients consumed regularly may inhibit platelet activation pathways [13]. More specifically, a number of dietary components including some fats, antioxidants and nucleosides have been shown to diminish platelet activation [14]. Regarding this, chlorogenic acid is one of the most abundant polyphenol compounds present in a variety of foods that are consumed daily, such as cherries, apples, kiwis, artichokes, eggplants, plums and coffee. Chlorogenic acid exhibits many biological properties, including antibacterial, antioxidant and anti-inflammatory activities, particularly hypoglycemic and hypolipidemic effects [15], [16], [17].

Chlorogenic acid is known for its antiplatelet activity resulting in cardiovascular protection [18], [19], although the specific mechanisms by which this inhibition occurs have not been fully established. In this study, we systematically examined the effects of chlorogenic acid on human platelets, and further characterize the detailed mechanisms of the chlorogenic acid-mediated inhibition of platelet activation. We report a multifaceted relationship between chlorogenic acid structure and anti-platelet effects.

## Materials and Methods

### Cell culture and Reagents

The HMEC-1 cell line was obtained from the Institute of Molecular Oncology, University of Milan, Milan, Italy. HMEC-1 was maintained routinely in a MCDB-131 medium (Gibco/Invitrogen) containing 10% heat-inactivated fetal bovine serum, 2 mmol/L glutamine, 1% antibiotic-antimycotic (Gibco/Invitrogen, USA), 1 µg/mL hydrocortisone (Sigma-Aldrich, St. Louis, Missouri/MO, U.S.A) and 10 ng/mL epithelial growth factor (Gibco/Invitrogen), at 37°C in a 5% CO_2_ humidified incubator.

Sodium chloride (p.a.) was obtained through Arquimed (Santiago, Chile). Adenosine 5′- diphosphate (ADP), thrombin receptor activator peptide 6 (TRAP-6), calcein-AM, collagen, arachidonic acid (AA), acetylsalicylic acid (ASA), cilostazol, bovine serum albumin (BSA), chlorogenic acid, SQ22536 (an adenylyl cyclase inhibitor), ZM241385 (A_2A_ receptor antagonist), protein kinase A (PKA) inhibitor H89, rose bengal, prostaglandin E_1_ (PGE_1_), dimethyl sulfoxide (DMSO), rhodamine 6G, fibrinogen and hepes were obtained from Sigma-Aldrich (St. Louis, Missouri/MO, U.S.A). Luciferase-luciferin reagent was obtained from Chrono-Log corp (Havertown, PA) and microfluidic chambers were sourced from Bioflux (Fluxion, San Francisco, California, USA). Anti-phospho-PKA antibody was obtained from Santa Cruz (Biotechnology, CA, USA). Anti γ-tubulin monoclonal antibody (4D11) was obtained from Thermo Scientific (Thermo Scientific, Pierce, Rockford, IL, USA). Antibodies (anti-CD62P-PE, anti-CD61-FITC, anti-GPIIb/IIIa-FITC PAC-1 and anti-CD61-PE) were obtained from BD Pharmingen (BD Biosciences, San Diego, CA, USA). DMSO 0.2% was employed as the vehicle.

### Preparation of washed platelets

After receiving written informed consent from all volunteers, venous blood samples were taken from six young healthy volunteers. The samples were placed in 3.2% citrate tubes (9∶1 v/v) by phlebotomy with vacuum tube system (Becton Dickinson Vacutainer Systems, Franklin Lakes, NJ, USA). The protocol was authorized by the ethics committee of the Universidad de Talca in accordance with the Declaration of Helsinki (approved by the 18th World Medical Assembly in Helsinki, Finland, 1964). Samples obtained from each volunteer were processed independently for each assay and centrifuged (DCS-16 Centrifugal Presvac RV) at 240 *g* for 10 min to obtain platelet-rich plasma (PRP). Following this, two-thirds of PRP was removed and centrifuged (10 min at 650 *g*). The pellet was then washed with HEPES-Tyrode's buffer containing PGE_1_ (120 nmol/L). Washed platelets were prepared in HEPES-Tyrode's buffer at a concentration of 200×10^6^ platelets/mL (Bayer Advia 60 Hematology System, Tarrytown, NY, USA). Platelets were kept at 4°C during all the isolation steps after blood samples were taken.

### Measurement of P-selectin surface expression and GPIIb/IIIa activation

Simultaneous measurements of P-selectin surface expression and glycoprotein (GP) IIb/IIIa activation were achieved by modifying methods previously described by Frojmovic *et al* [20]. Briefly, 480 µL of washed platelets at a concentration of 200×10^6^ platelets/mL were preincubated with 20 µL of vehicle or chlorogenic acid (0.1 to 1 mmol/L) for 3 min. After 6 min of stimulation at 37°C with ADP 8 µmol/L, the platelets were kept at 4°C. To determine platelet P-selectin expression, 50 µL of the sample was mixed with saturated concentrations of anti- CD62P-PE and anti-CD61-FITC and incubated for 25 min in the dark. To determine platelet GPIIb/IIIa expression, 50 µL of the sample was mixed with saturated concentrations of activation-specific anti-GPIIb/IIIa antibody PAC-1 and anti-CD61-PE and incubated for 25 min in the dark. After quench-dilution (≈4-fold), the samples were analyzed in an Accuri C6 flow cytometer (BD, Biosciences, USA). Platelet populations were gated on cell size using forward scatter (FSC) vs. side scatter (SSC) and CD61 positivity to distinguish from electronic noise. Light scatter and fluorescence channels were set at logarithmic gain and 5000 events per sample were analyzed. Fluorescence intensities of differentially stained populations were expressed as a mean channel value using BD Accuri C6 Software (BD Biosciences, USA). All measurements were performed from six separate platelet donors.

### Measurement of platelet secretion and aggregation

Platelet secretion and aggregation were determined by measuring ATP release (luciferin/luciferase reagent), and light transmission in a lumi-aggregometer (Chrono-Log, Havertown, PA, USA), respectively. Luciferin/luciferase (50 µL) was added to 480 µL of the washed platelets (adjusted to 200×10^6^ platelets/mL) within 2 min before stimulation. Secretion and aggregation were analyzed after preincubation of platelets with 20 µL of vehicle, ASA (0.3 mmol/L) or chlorogenic acid (0.05 to 1 mmol/L) for 3 min prior to the addition of ADP 8 µmol/L, collagen 1.5 µg/mL, TRAP-6 30 µmol/L or AA 1 mmol/L in the presence of fibrinogen (275 µ/mL). Platelet secretion and aggregation were measured at real time over a 6 min period at 37°C while stirring (150* g*). All measurements were carried out from six separate platelet donors. Chlorogenic acid inhibition of the maximal platelet secretion and aggregation were expressed as a percentage of the vehicle (DMSO 0.2%). The concentration required to inhibit platelet secretion and aggregation by 50% (IC_50_) was calculated from the dose-response curves.

### Platelet rolling and firm adhesion under controlled flow conditions

Experiments under flow-controlled conditions were performed in a BioFlux-200 flow system (Fluxion, San Francisco, California, USA) with high shear plates (48 wells, 0-20 dyne/cm^2^) [21]. The microfluidic chambers were coated with 20 µL of collagen (200 µg/mL) at a wall shear rate of 2 dyne/cm^2^ for 1 hour. The plaque coating was allowed to dry at room temperature (RT) for one hour. Thereafter, channels were perfused with phosphate buffered saline (PBS) for 10 min to remove the interface and blocked with BSA 5% for 10 min (RT, wall shear rate 2 dyne/cm^2^). Whole blood, anticoagulated with sodium citrate, was labeled with calcein-AM (4 µmol/L) and incubated with vehicle, ASA (0.3 mmol/L) or chlorogenic acid (0.1 to 1 mmol/L) for 15 min (RT) before being added into the inlet of the well. Chambers were perfused for 10 min at RT at a wall shear rate of 10 dyne/cm^2^. The plates were mounted on the stage of an inverted fluorescence microscope (TE200, NIKON, Japan).

Platelet deposition was observed and recorded at real-time with a CCD camera (QICAM, QIMaging, Surrey, BC, Canada). Real-time visualization between platelets and collagen interactions were performed using a bright field and fluorescence microscopy. For each flow experiment, fluorescence images were analyzed off-stage by quantifying the area covered by platelets with ImageJ software (version 1.26t, NIH, USA). In each field, the area covered by platelets was quantified. All measurements were performed from six separate platelet donors.

### Leukocyte rolling over collagen-bound platelet monolayer under controlled flow

The collagen-coated microfluidic chambers were rinsed in PBS buffer and perfused with whole blood (anticoagulated with sodium citrate) labeled with calcein-AM 4 µmol/L at a shear rate of 2 dyne/cm^2^ for 10 min at RT. Under these conditions, a homogeneous carpet of spread platelets was formed. The remaining blood was washed with vehicle, ASA (0.3 mmol/L) or chlorogenic acid (0.1 to 1 mmol/L) for 3 min. Subsequently, leukocytes rolling and firm adhesion were visualized on the platelet layer with an inverted fluorescence microscope. Leukocytes were previously labeled with rhodamine 6G (50 µl of 0.05%). Digital images were captured and translocation velocity was calculated by image analysis (Image J) [22]. All measurements were performed from six separate platelet donors.

### Measurement of cAMP levels in platelets

The effect of chlorogenic acid (0.1 to 1 mmol/L) on cAMP platelet levels was evaluated in 480 µL of washed platelets (200×10^6^ platelets/mL) after a 5-min incubation period without stirring. Platelet reaction was stopped in cold-ice 10% trichloroacetic acid and precipitated proteins were removed by centrifugation. Following this, HCl 1 mol/L (150 µl) was added and supernatants were subjected to 6 ether extractions (v/v) and lyophilized. Samples were stored at −70°C until analysis. Before determination, samples were dissolved in 200 µL PBS at a pH 6.2. cAMP, Direct Immunoassay Kit (BioVision Research Products, Mountain View, CA, USA) was performed. All measurements were performed from six separate platelet donors.

### Platelet inflammatory mediators

Soluble CD40 ligand (sCD40L), CCL5 and IL-1β were determined using human quantikine ELISA kits (R&D systems, Minneapolis, MN) and soluble P-selectin levels (sP-selectin) was determined by ELISA according to the manufacturer's instructions (Invitrogen Corporation, California, USA). Briefly, washed platelets were pretreated with vehicle, ASA (0.3 mmol/L) or chlorogenic acid (0.1 to 1 mmol/L) for 15 min at 37°C and then stimulated with thrombin (2 U/mL) for 45 min at 37°C. Finally, the supernatants were collected after centrifugation (2.000 *g,* 10 min, 4°C) and stored at −70°C until use. All measurements were performed from six separate platelet donors.

### Effect of SQ22536 and ZM241385 on antiplatelet activity of chlorogenic acid

Washed platelets (480 µL at 200×10^6^ platelets/mL) were pretreated with SQ22536 (200 and 400 µmol/L) or ZM241385 (15 and 30 µmol/L) for 3 min before the addition of chlorogenic acid (1 mmol/L). Then, platelet aggregation was challenged by ADP 8 µmol/L. Platelets were first exposed to SQ22536 or ZM241385 and then ADP was used as controls. Moreover, washed platelets were pretreated with SQ22536 (200 and 400 µmol/L) and then with vehicle or chlorogenic acid (1 mmol/L) for 15 min at 37°C before thrombin (2 U/mL) stimulation (45 min at 37°C). sP-selectin and sCD40L release were assessed as described above. All measurements were performed from six separate platelet donors.

### Western blotting

Washed platelets (200×10^6^ platelets/mL) were pre-incubated with vehicle, chlorogenic acid (0.1 to 1 mmol/L), or PKA inhibitor H89 (50 µmol/L) and chlorogenic acid (1 mmol/L). Then, platelets were lysed with 0.2 mL of lysis buffer in ice for 30 min and heated for 10 min at 95°C. Equal quantities of total protein (30 µg) were subjected to SDS-PAGE under reducing conditions and transferred to a nitrocellulose membrane. The proteins were detected with anti-phospho-PKA and anti-γ-tubulin antibodies. All measurements were performed from six separate platelet donors.

### Cytotoxicity assay

Due to the short platelet half-life of, an endothelial cell line was employed for cytotoxicity studies. The effect of chlorogenic acid on endothelial cell viability was assessed using a Vybrant MTT Cell Proliferation Assay Kit (Invitrogen). Briefly, confluent cells were seeded in 96-well plates after a 4-hour chlorogenic acid treatment (0.05 to 1 mmol/L) and incubated for 3 hours with 3-(4,5-dimethylthiazol-2-yl)-2,5-diphenyltetrazolium bromide (MTT) 12 mmol/L [23]. Then, SDS-HCl solution was added to each well and after 16 hours, the absorbance was measured at 570 nm (BioTeK Synergy H1 Hybrid Reader). Cells treated with medium only were used as a negative control group. Cell viability was expressed as a percentage relative to the untreated control cells.

### *In vivo* murine model of thrombosis

This study was carried out under recommendations by the Guide for the Care and Use of Laboratory Animals of the National Institutes of Health. The protocol was approved by the Committee on the Ethics of Animal Experiments of the University of Talca. All efforts were made to minimize suffering. Thrombosis in mice was performed by photochemical injury using modified methods described by Przyklenk and Whittaker [24]. Briefly, C57BL/6 mice (12–16 weeks old) were anesthetized with a combination of tribromoethanol (270 mg/kg) and xylazine (13 mg/kg). The mesentery was exposed by performing a central incision at the abdomen, permitting the visualization of thrombus development in mesenteric vessels by intravital microscopy. Thrombosis was induced by an injection of 50 mg/kg rose bengal through the tail vein followed by illumination of the exposed mesenteric area with a 1.5-mW green light laser (532 nm). Blood flow was monitored for 60 min and stable occlusion was defined as a blood flow of 0 mL/min for 3 min. Vehicle (control group, n = 6), ASA (200 mg/kg, n = 6) or chlorogenic acid (200 mg/kg, n = 6) were administered intraperitoneally 30 min before the experiment. Rectal temperatures were similar and within the physiological range between all experimental animals throughout the experimental period.

### Bleeding Assay

C57BL/6 mice were anesthetized with a combination of tribromoethanol (270 mg/kg) and xylazine (13 mg/kg) and placed prone on a warming pad from which the tail protruded. The same amounts of chlorogenic acid (200 mg/kg, n = 6, intraperitoneally), ASA (200 mg/kg, n = 6, intraperitoneally) or vehicle (DMSO 0.2%, n = 6, intraperitoneally) as described in the thrombosis model were given. An incision was made on the ventral surface of the rat tails about 2 mm from the tip [25]. The bleeding time was measured in seconds (s) until bleeding stopped.

### Molecular Docking

Docking was performed using Glide [26] contained in the Maestro software (Maestro, Version 9.0, Schrödinger, LLC, New York, NY, 2007). Glide docking uses a series of hierarchical filters to find the best possible ligand binding locations in a previously built receptor grid space. The filters include a systematic search approach which samples the positional, conformational, and orientational space of the ligand before evaluating the energy interactions between the ligand and the protein.

The coordinates of the adenosine receptor A_2A_ were extracted from the X-ray crystal structure of the human A_2A_ receptor with adenosine bound (accession code in Protein Data Bank (PDB): 2YDO). The structure of the chlorogenic acid was sketched with Maestro software. The extra-precision (XP) module of Glide was employed. A grid box of 30 Å × 30 Å × 30 Å was first centered on the center of mass of the adenosine in PDB 2YDO and default docking parameters were used [27], [28]. The docking hierarchy begins with the systematic conformational expansion of the ligand followed by placement in the receptor site. Then minimization of the ligand in the receptor field was carried out using the OPLS-AA [29] force field with a distance-dependent dielectric of 2.0. Afterwards, the lowest energy poses were subjected to a Monte Carlo procedure that samples the nearby torsional minima. The best pose for a given ligand was determined by the Emodel score, while different compounds were ranked using GlideScore [30]. The docking poses for both ligands were analyzed by examining their relative total energy score. The more energetically favorable conformations were selected as the best poses.

### Statistical analysis

Data were analyzed using SPSS version 17.0 (SPSS, Inc., Chicago, Illinois) and expressed as mean ± standard error of mean (SEM). All measurements were performed from six separate platelet donors. Results were expressed as percent inhibition or as percentage of vehicle (as 100%). Fifty-percent inhibitory concentration of chlorogenic acid against agonist-induced platelet activation was calculated from the dose-response curves. Differences between groups were analyzed by student paired or unpaired t-test and one-way analysis of variance (ANOVA) using Tukey's post-hoc test. P values <0.05 were considered significant.

## Results

### Effects of chlorogenic acid on P-selectin expression and GPIIb/IIIa activation

**Figure 1:**
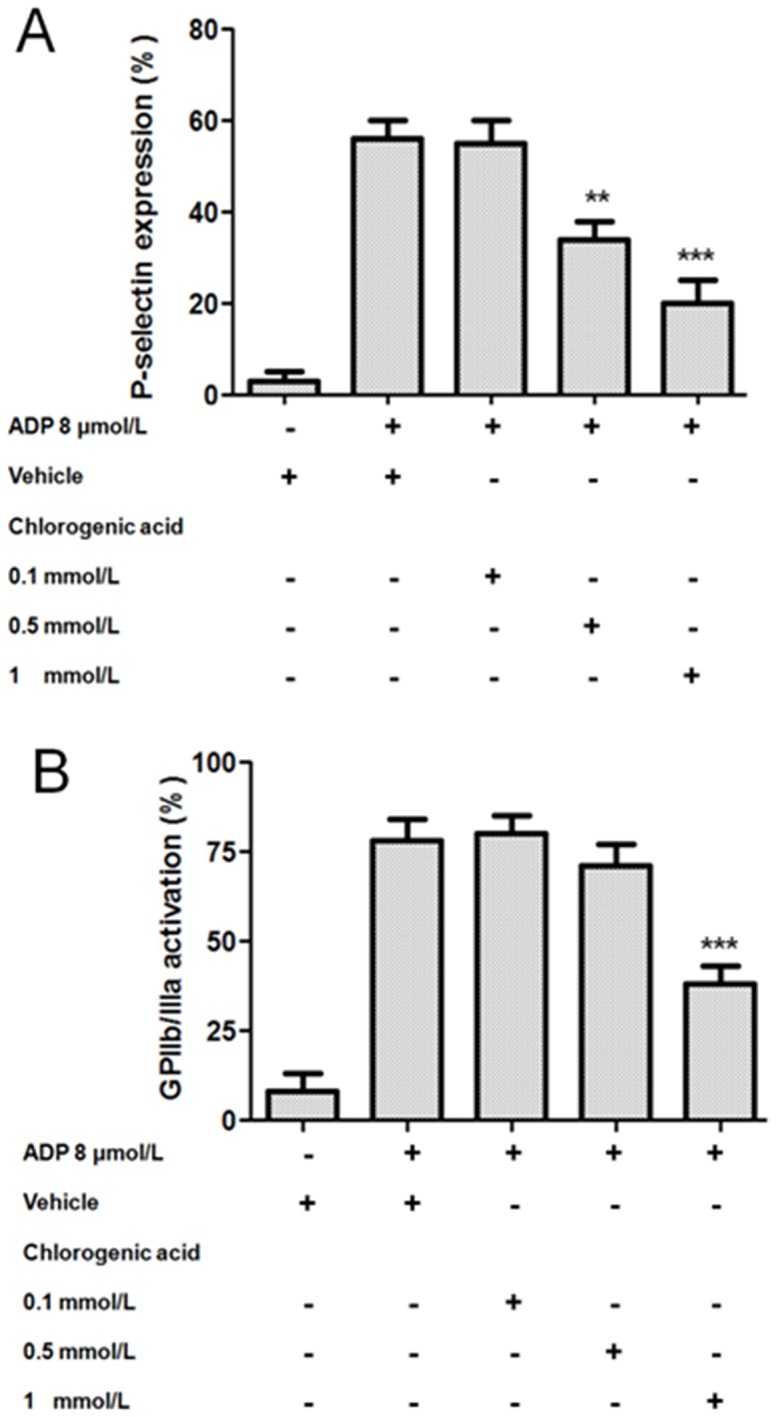
Effects of chlorogenic acid on P-selectin expression and GPIIb/IIIa activation. Washed platelets were preincubated with vehicle (DMSO 0.2%) or chlorogenic acid (0.1 to 1 mmol/L) for 3 min. Then, samples were treated for 6 min with ADP 8 µmol/L. P-selectin expression (A) and GPIIb/IIIa activation (B) were determined by flow cytometry. Results were expressed as mean ± SEM, n = 6. **p<0.01 and ***p<0.001. The results presented are from 6 separate volunteers.

After platelet activation, P-selectin was translocated from intracellular granules to the external membrane, whereas the fibrinogen causes platelets to aggregate by bridging GPIIb/IIIa between adjacent platelets [31]. As shown in figure 1A, ADP induced P-selectin expression on platelets was decreased by chlorogenic acid. P-selectin expression in the presence of chlorogenic acid (0.5 and 1 mmol/L) was inhibited by 39±4 (p<0.01) and 64±4% (p<0.001), respectively. Moreover, chlorogenic acid at 1 mmol/L only inhibited ADP-induced platelet GPIIb/IIIa activation by 51±5% (p<0.001) (figure 1B). In this way, chlorogenic acid inhibited platelet activation by decreasing P-selectin expression and lowering GPIIb/IIIa activation.

### Effects of chlorogenic acid on platelet ATP secretion and aggregation

**Figure 2:**
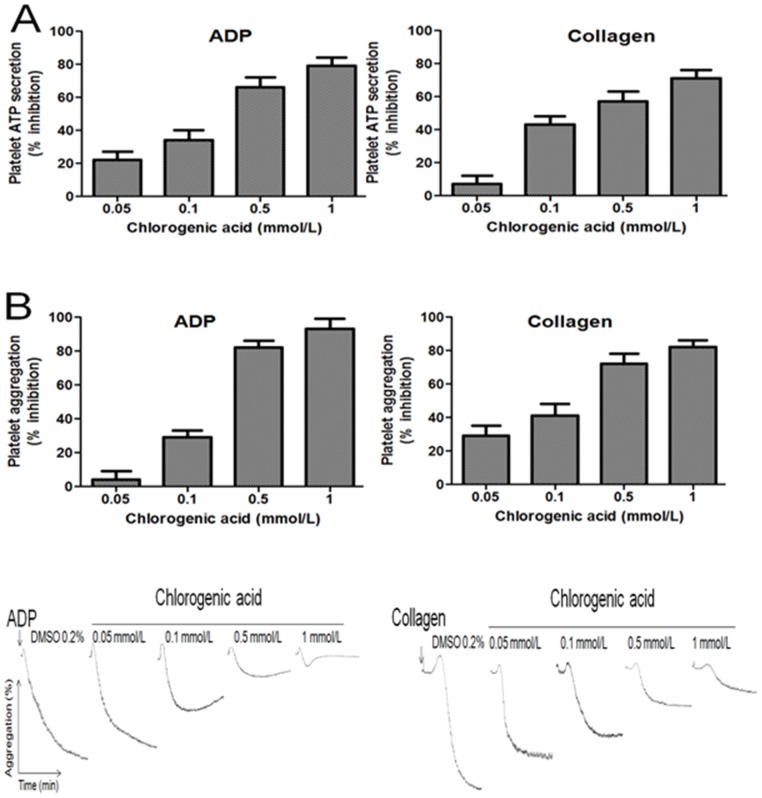
Effects of chlorogenic acid on platelet ATP secretion (A) and aggregation (B). For A) ADP 8 µmol/L (thromboxane A2 dependent) and collagen 1.5 µg/mL induced platelet ATP secretion. For B) platelet aggregation induced by ADP 8 µmol/L and collagen 1.5 µg/mL and each aggregation curve is representative of multiple traces obtained from six separate platelet donors. Results were expressed as % inhibition (mean ± SEM, n = 6).

The effects of chlorogenic acid on platelet ATP-secretion and aggregation induced by ADP and collagen are shown in figure 2. Chlorogenic acid inhibited ADP-induced ATP secretion, with a calculated IC_50_ concentration of 0.41 mmol/L. Similarly, the IC_50_ concentration for chlorogenic acid on collagen-induced platelet ATP-secretion was 0.51 mmol/L. Chlorogenic acid at a concentration of 1 mmol/L showed a mild inhibitory effect (19±4 and 23±5%, p<0.05) on TRAP-6 and AA-induced platelet ATP-secretion. Therefore, chlorogenic acid inhibited platelet aggregation induced by ADP and collagen, but to a different extent.

In addition, chlorogenic acid effectively reduced ADP-induced platelet aggregation with an IC_50_ of 0.39 mmol/L. Similarly; chlorogenic acid suppressed collagen-induced platelet aggregation with an IC_50_ of 0.30 mmol/L. Moreover, at a concentration of 1 mmol/L chlorogenic acid showed a mild inhibitory effect (26±5 and 32±4%, p<0.05) on TRAP-6 and AA-induced platelet aggregation. Consequently, chlorogenic acid inhibited platelet ATP secretion induced by ADP and collagen, but to a different extent.

### Chlorogenic acid impairs platelet adhesion on immobilized collagen under flow conditions

**Figure 3:**
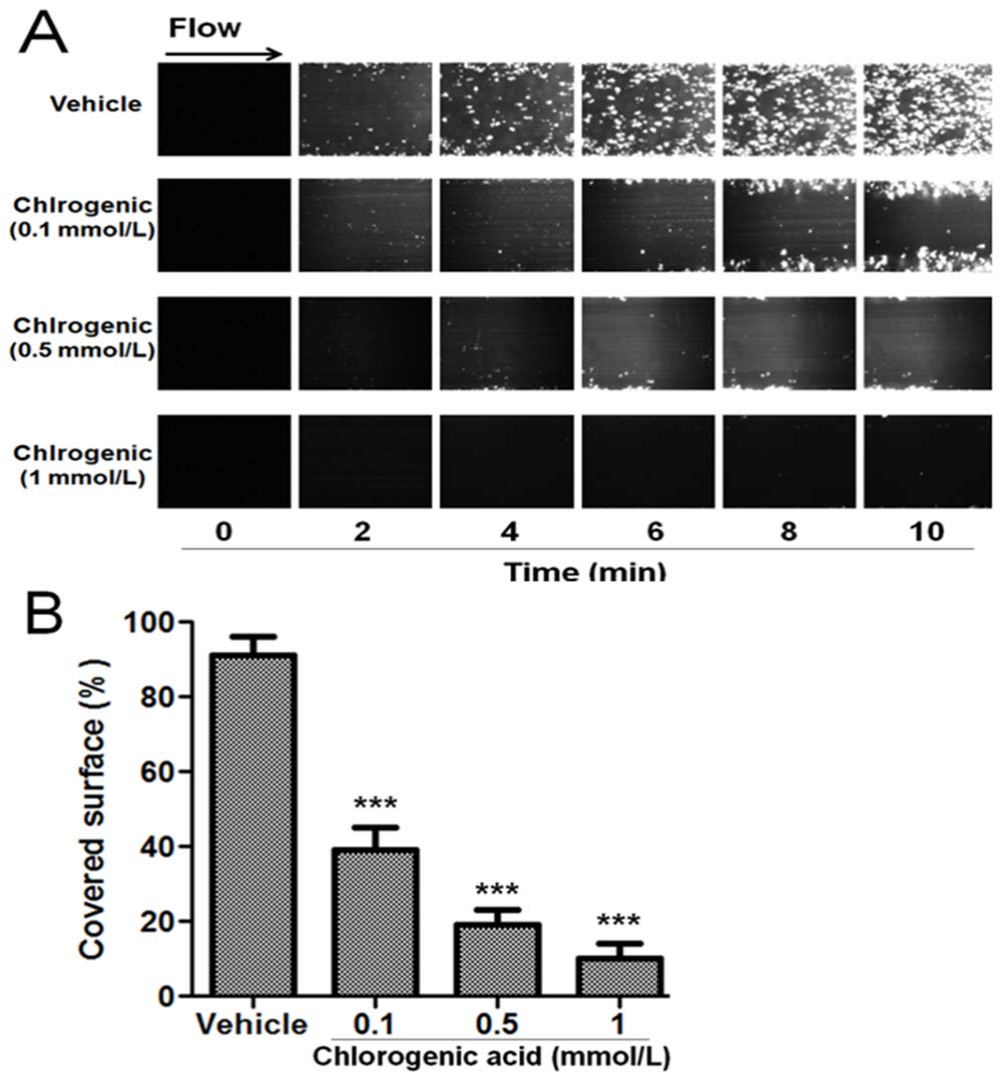
Effects of chlorogenic acid on collagen-induced platelet adhesion and aggregation under arterial flow conditions. Citrate-anticoagulated blood was pre-incubated with vehicle (DMSO 0.2%) or chlorogenic acid (0.1 to 1 mmol/L) for 15 min and then was perfused over plaque-coated surfaces for 10 min at room temperature at a shear rate of 10 dyne/cm^2^. A) Timelapse of 10 min at 10 dyne/cm^2^, at 2 min intervals. B) Surface covered by platelets expressed as the percentage of the total surface observed, values are mean ± SEM; n = 6. ***p<0.001. The results presented are from 6 separate volunteers.

Rapid platelet adhesion and aggregate formation were observed after perfusion of citrate-anticoagulated blood over collagen-coated plate surfaces at 37°C with a wall shear rate of 10 dyne/cm^2^ for 10 min (figure 3A). In the presence of chlorogenic acid (0.1 to 1 mmol/L) platelet firm adhesion and aggregation on the collagen-surface was significantly lower compared to control (figure 3B). After blood chlorogenic acid incubation, platelet coverage was inhibited by 57±6, 79±4 and 89±4% at concentrations of 0.1, 0.5 and 1 mmol/L, respectively (p<0.001). Therefore, chlorogenic acid reduced collagen-induced platelet adhesion and aggregate formation under flow-controlled conditions.

### Effects of chlorogenic acid on platelet-leukocyte interactions

**Figure 4:**
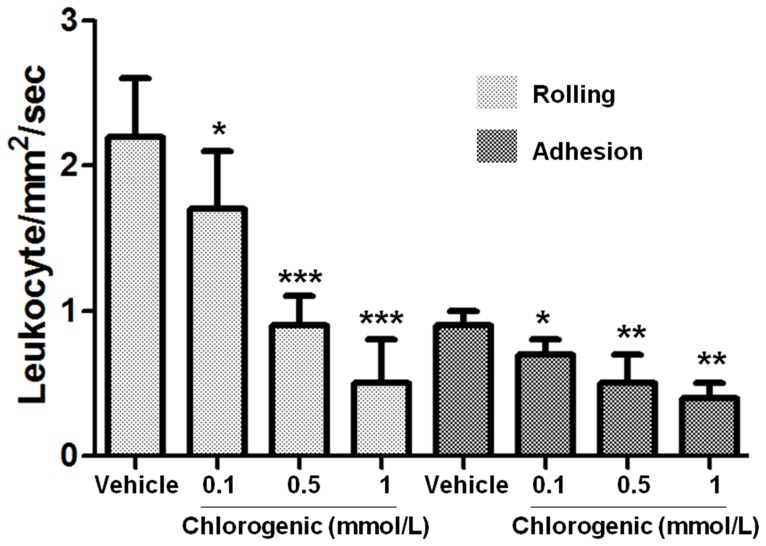
Chlorogenic acid reduces leukocyte rolling and firm adhesion over collagen-bound platelet monolayers. Velocities of leukocyte rolling and firm adhesion on platelet monolayer surface in presence of chlorogenic acid. Results were expressed as mean ± SEM of n = 6. The significant results (*p<0.05, **p<0.01 and ***p<0.001) are between vehicle group (DMSO 0.2%) and chlorogenic acid dose. The results presented are from 6 separate volunteers.

The effects of chlorogenic acid on platelet-leukocyte interactions are shown in figure 4. Under shear stress of 2 dyne/cm^2^, leukocytes rolled and attached to activated platelets but not to collagen surfaces (data not shown). Chlorogenic acid attenuated in concentration-dependent manner (0.1 to 1 mmol/L) interactions between leukocytes and platelet surface, reflecting a reduction of rolling and lower adhesion. As shown in figure 4, rolling velocity of leukocytes under the shear stress of 2 dyne/cm^2^ over immobilized activated platelets was diminished in the presence of chlorogenic acid 0.1, 0.5 and 1 mmol/L from 2.2±0.4 in the control group to 1.7±0.4 (p<0.05), 0.9±0.2 (p<0.001) and 0.5±0.3 (p<0.001) mm^2^/s, respectively. Similarly, firm adhesion of leukocyte immobilized activated platelets was diminished in the presence of chlorogenic acid 0.1, 0.5 and 1 mmol/L from 0.9±0.1 in the control group to 0.7±0.1 (p<0.05), 0.5±0.2 (p<0.01) and 0.4±0.1 (p<0.01) mm^2^/s, respectively. In this way, chlorogenic acid reduced platelet-leukocyte interactions under flow conditions.

### Effects of chlorogenic acid on intraplatelet levels of cAMP

**Figure 5:**
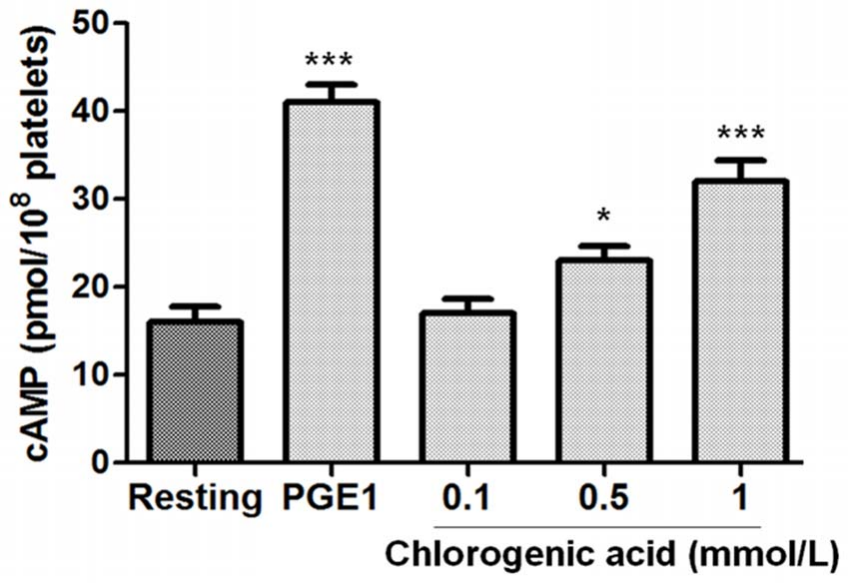
Effect of chlorogenic acid on intraplatelet levels of cAMP formation in human platelets. Platelets were incubated with PGE_1_ (0.02 mmol/L, positive control) or chlorogenic acid (0.1 to 1 mmol/L) for measurement of cAMP formations as described in “Materials and Methods.” Results were expressed as mean ± SEM of n = 6. The significant results (*p<0.05 and ***p<0.001) are between resting group, and PGE1 and chlorogenic acid dose. The results presented are from 6 separate volunteers.

We investigated whether chlorogenic acid effects on platelet function were mediated by changes of intraplatelet levels of cAMP. As shown in figure 5, chlorogenic acid inhibition of platelet aggregation was associated with an increase of cAMP intraplatelet levels (p<0.001). As a control for the assay, levels of cAMP in resting platelets were marked lower than those observed in PGE1 (0.02 mmol/L)-treated platelets (p<0.001) (figure 5).

### Effects of chlorogenic acid on platelet inflammatory mediators

**Figure 6:**
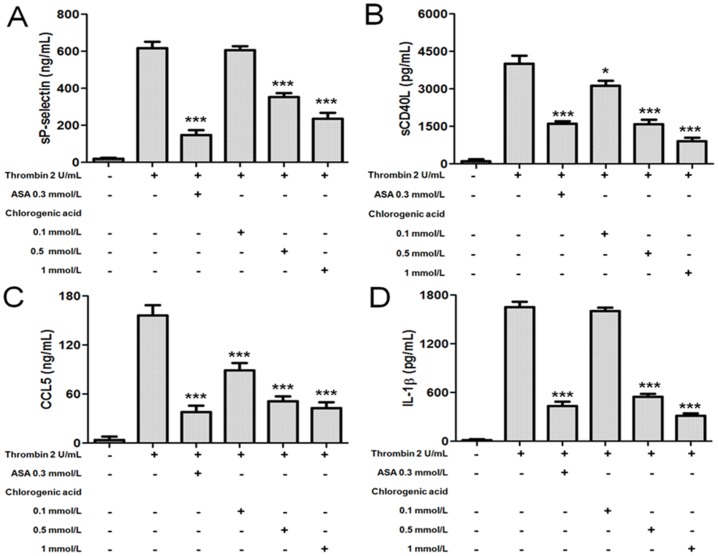
Effects of chlorogenic acid on platelet sP-selectin, sCD40L, CCL5 and IL-1β release. Washed platelets were pretreated with vehicle (DMSO 0.2%), ASA (0.3 mmol/L) or chlorogenic acid (0.1 to 1 mmol/L) for 15 min at 37°C and then stimulated by thrombin (2 U/mL). The graph depicts the mean ± SEM of n = 6 experiments. *p<0.05 and *** p<0.001. The results presented are from 6 separate volunteers.

Pretreatment of washed platelets with increasing concentrations of chlorogenic acid (0.1 to 1 mmol/L) significantly inhibited thrombin-induced sP-selectin, sCD40L, CCL5 and IL-1β release (figure 6). Thus, thrombin-induced sP-selectin release was inhibited by 42±4 and 61±3%, (p<0.001) in the presence of chlorogenic acid at 0.5 and 1 mmol/L, respectively (figure 6A). Moreover, we examined the effect of chlorogenic acid on platelet sCD40L release. As observed in figure 6B, we found that chlorogenic acid significantly reduced thrombin-induced platelet sCD40L release by 22±2 (p<0.05), 60±3 (p<0.001) and 77±4% (p<0.001) at concentrations of 0.1, 0.5 and 1 mmol/L, respectively. Also, this study shows that chlorogenic acid inhibited two important platelet-derived inflammatory mediator releases after activation, both CCL5 chemokine and IL-1β. In this study, chlorogenic acid 0.1, 0.5 and 1 mmol/L attenuated the effect of thrombin-induced CCL5 release from 156±13 in the control group to 89±9, 51±6 and 43±7 (p<0.001) ng/mL, respectively (figure 6C). Meanwhile, thrombin-induced IL-1β release was diminished in the presence of chlorogenic acid at 0.5 and 1 mmol/L from 1650±67 in the control group to 547±39 and 312±31 (p<0.001) pg/mL (figure 6D). Thus, we have demonstrated that chlorogenic acid inhibited platelet inflammatory mediators (sP-selectin, sCD40L, CCL5 and IL-1β release).

### SQ22536 and ZM241385 attenuated the effect of chlorogenic acid on platelet activation

**Figure 7:**
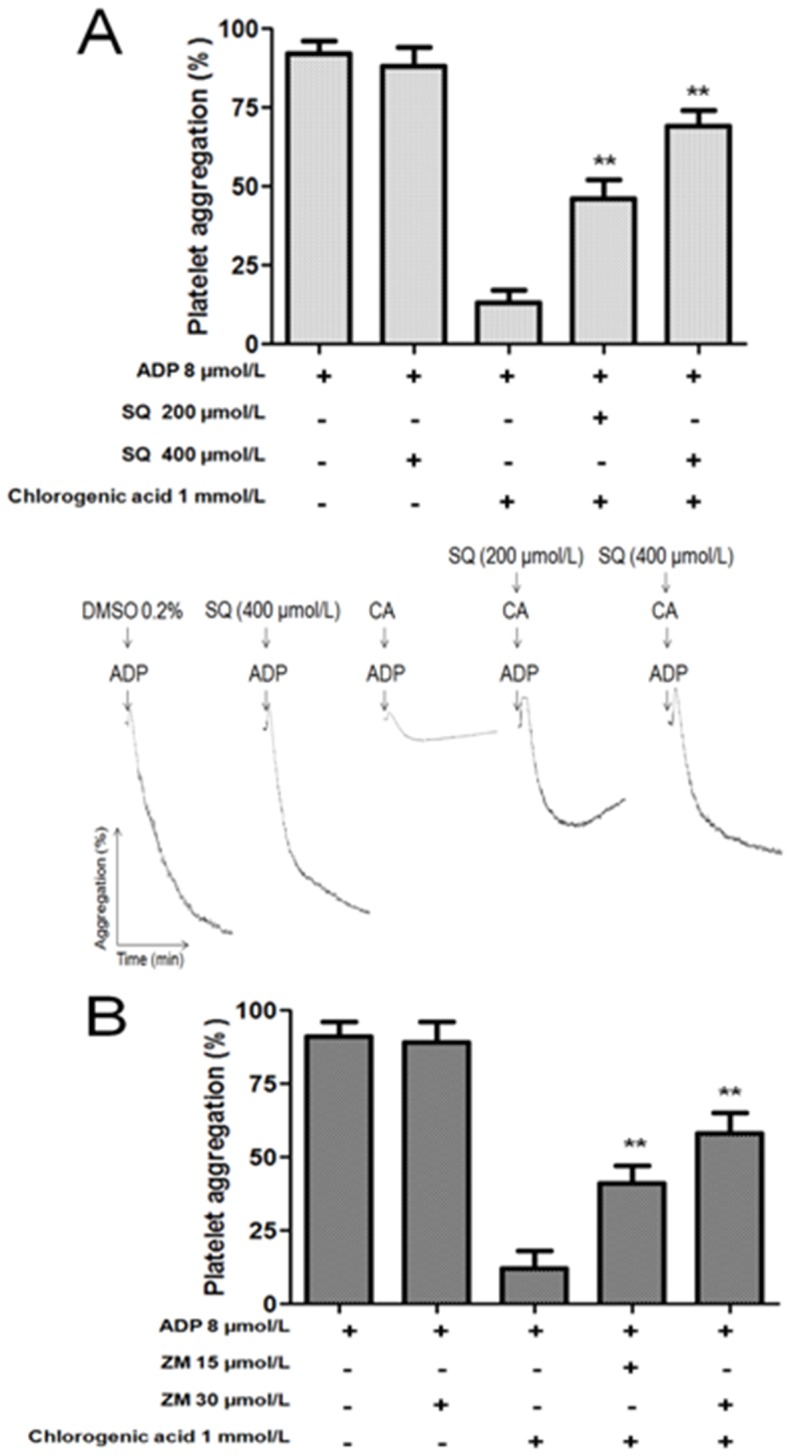
Effects of SQ22536 and ZM241385 on antiplatelet activity of chlorogenic acid. For platelet aggregation washed platelets suspension was incubated with ADP, chlorogenic acid plus ADP or pretreated with SQ22536 (A) or ZM241385 (B) for 3 min, followed by addition of chlorogenic acid (CA) and ADP. The graph depicts the mean ± SEM of n = 6 experiments. ** p<0.01. The results presented are from 6 separate volunteers.

In this study, we found that SQ22536, an adenylyl cyclase inhibitor, could reverse the inhibitory effects of chlorogenic acid on platelet aggregation induced by ADP. As shown in figure 7A, SQ22536 attenuated the inhibitory effect of chlorogenic acid against ADP-induced platelet aggregation by 36±3 and 61±4% at concentrations of 200 and 400 µmol/L, respectively (p<0.01). Moreover, we tested whether ZM241385, a potent A_2A_ receptor antagonist, could reverse the inhibitory effect of chlorogenic acid on platelet aggregation induced by ADP. Therefore, ZM241385 attenuated the inhibitory effect of chlorogenic acid against ADP-induced platelet aggregation by 31±3 and 50±4% at concentrations of 15 and 30 µmol/L, respectively (p<0.01) (figure 7B). Antiplatelet activity of chlorogenic acid was inhibited by both SQ22536 and ZM241385. As a control, SQ22536 (400 µmol/L) and ZM241385 (30 µmol/L) alone did not exert any effect on ADP (8 µmol/L)-induced platelet aggregation.

**Figure 8:**
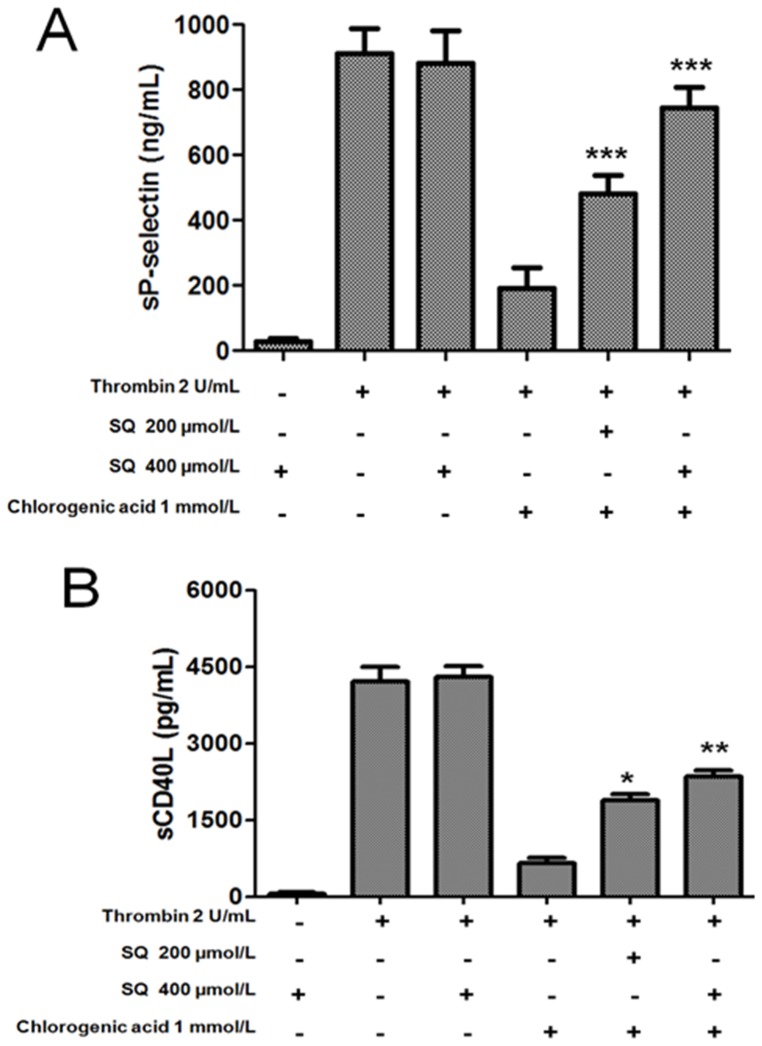
Effects of SQ22536 on mechanism of antiplatelet action of chlorogenic acid. For sP-selectin (A) and sCD40L (B) washed platelets were incubated with thrombin, chlorogenic acid plus thrombin or pretreated with SQ22536 for 3 min, followed by the addition of chlorogenic acid and thrombin. The graph depicts the mean ± SEM of n = 6 experiments. * p<0.05, ** p<0.01 and *** p<0.001. The results presented are from 6 separate volunteers.

Moreover, SQ22536 (200 and 400 µmol/L) reverted the chlorogenic acid inhibitory effect on platelet sP-selectin release (thrombin-induced) by 32±3 and 61±4%, respectively (p<0.01). In addition, SQ22536 (200 and 400 µmol/L) could reverse the inhibitory effect of chlorogenic acid on platelet sCD40L release (thrombin-induced) by 28±3 (p<0.05) and 40±4% (p<0.01), respectively (figure 8). SQ22536 400 µmol/L alone did not exert any effect on thrombin-induced sP-selectin and sCD40L release.

### PKA activation by chlorogenic acid

**Figure 9:**
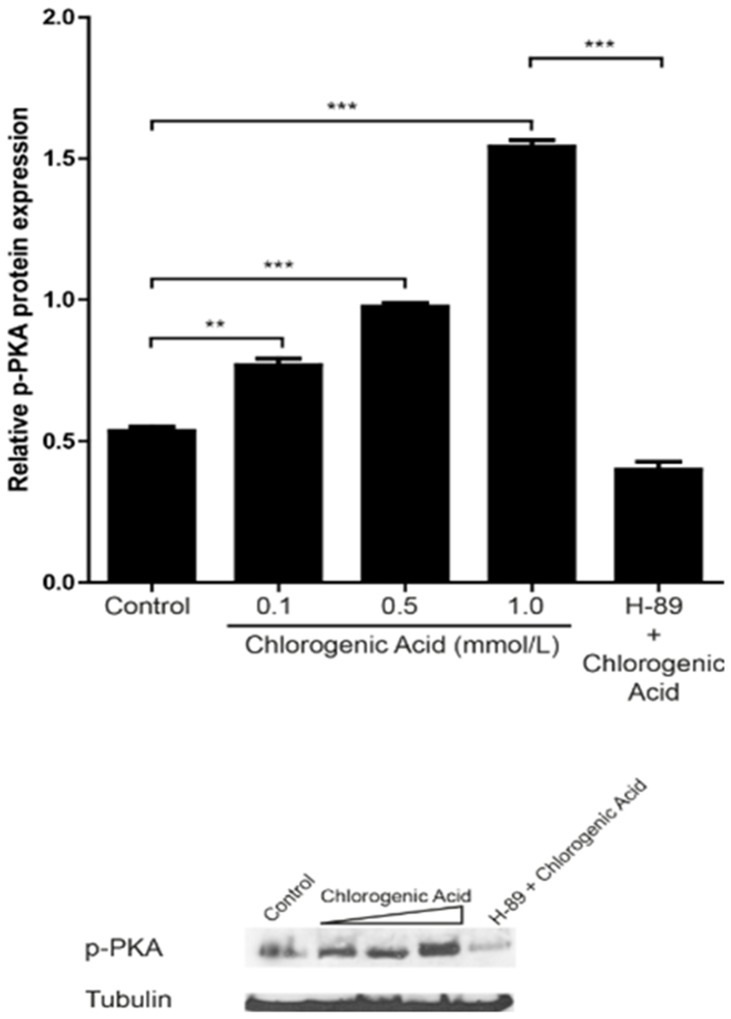
Effect of chlorogenic acid on PKA activation. Platelets were collected and subcellular extracts were analyzed for phospho-PKA as described in Materials and Methods. Data are presented as the mean ± SEM of n = 6 experiments. **p<0.01 and *** p<0.001. The results presented are from 6 separate volunteers.

PKA activation plays a vital role in maintaining circulating platelets in a resting state. Here, we show that the treatment of washed platelets with chlorogenic acid (0.1 to 1 mmol/L) markedly increased the phosphorylation of PKA in resting platelets (figure 9).

### Effect of chlorogenic acid on cell viability

To discount a cytotoxic effect, chlorogenic acid was evaluated at the same concentrations that inhibited platelet function by basal cytotoxicity (MTT assay) using HMEC-1 cell line. Chlorogenic acid at 0.05 to 1 mmol/L showed no cytotoxicity effect with cell viability over 90%.

### Effect of chlorogenic acid on arterial thrombus formation *in vivo*

**Figure 10:**
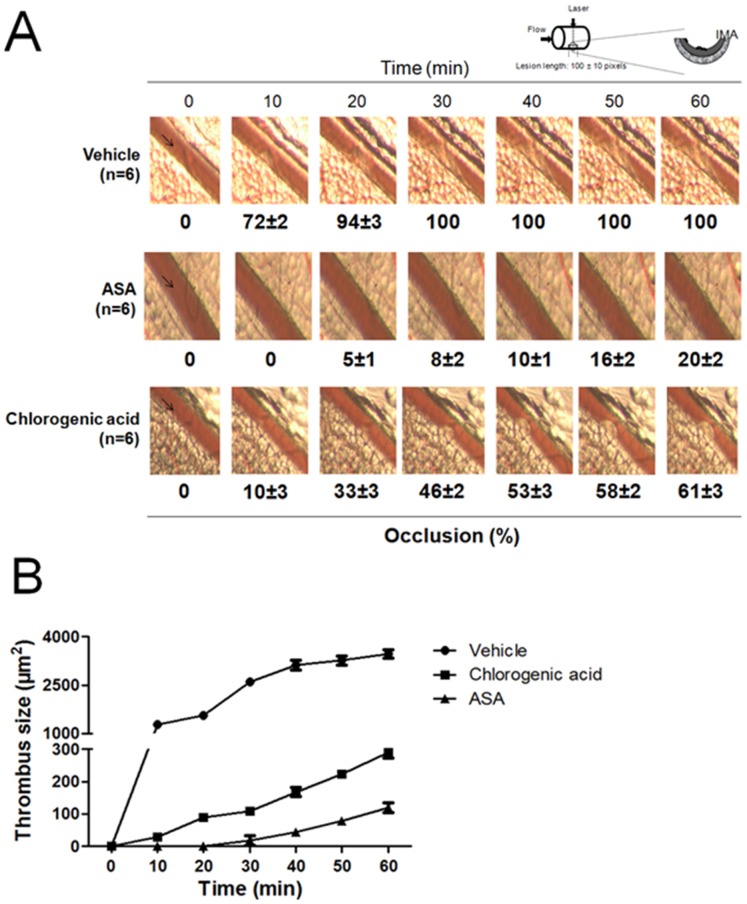
Chlorogenic acid inhibited arterial thrombosis formation. A) Representative images of thrombus formation after laser irradiation in the vehicle group (DMSO 0.2%, n = 6); ASA (200 mg/kg, n = 6) or chlorogenic acid (200 mg/kg, n = 6) to 60 min. B) Time course changes of thrombus growth rate. I, intima; M, media and A, adventitia.

As shown in figure 10, the mesenteric vessel of untreated mice (control) was completely occluded by a stable bulky thrombus 30 min after laser injury (>2500 µm^2^). In contrast, one intraperitoneal bolus injection of chlorogenic acid (200 mg/kg) 30 min before laser injury prolonged the time to vessel occlusion to 60 min and reduced the maximum occlusion to 61±3%. Therefore, chlorogenic acid possesses antithrombotic activity.

### Effects of chlorogenic acid on bleeding time

**Figure 11:**
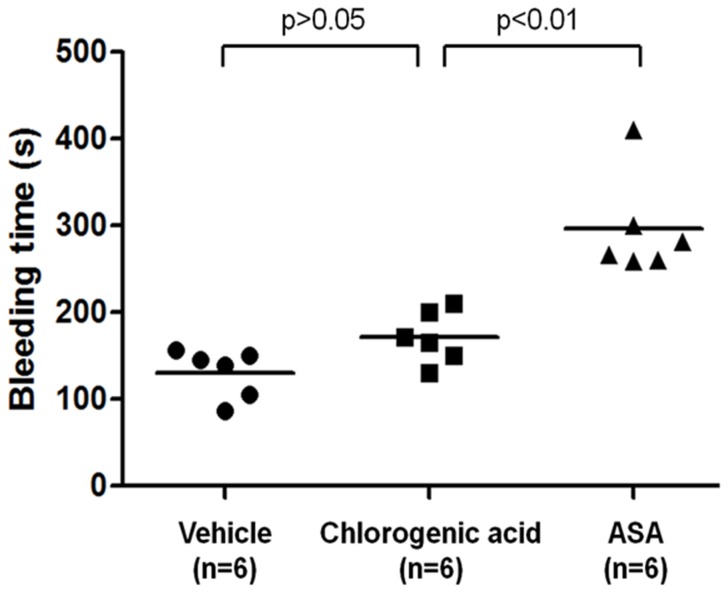
Effect of chlorogenic acid on bleeding time. Same amount of vehicle (DMSO 0.2%, n = 6, intraperitoneally), chlorogenic acid (200 mg/kg, n = 6, intraperitoneally) or ASA (200 mg/kg, n = 6, intraperitoneally) as in thrombosis model were given. Each dot represents the bleeding time measured in each individual mouse (n = 6), mean is also shown.

To test the possible bleeding side effects of chlorogenic acid, we measured chlorogenic acid-induced C57BL/6 mouse blood loss after tail snip at the same concentration (200 mg/kg, a single bolus intraperitoneally injection) that was used for *in vivo* antithrombotic study. The bleeding time by chlorogenic acid was 176±33 s (n = 6), which was higher but not statistically significantly higher than the vehicle (135±21 s, n = 6) (p>0.05). In addition, compared with ASA (ASA  =  281±43 s, n = 6), chlorogenic acid was significantly lower (p<0.01) (figure 11). Therefore, at the antithrombotic concentration used, chlorogenic acid did not cause significant bleeding measured by tail snip.

### Molecular modeling of chlorogenic acid on A_2A_ receptor

**Figure 12:**
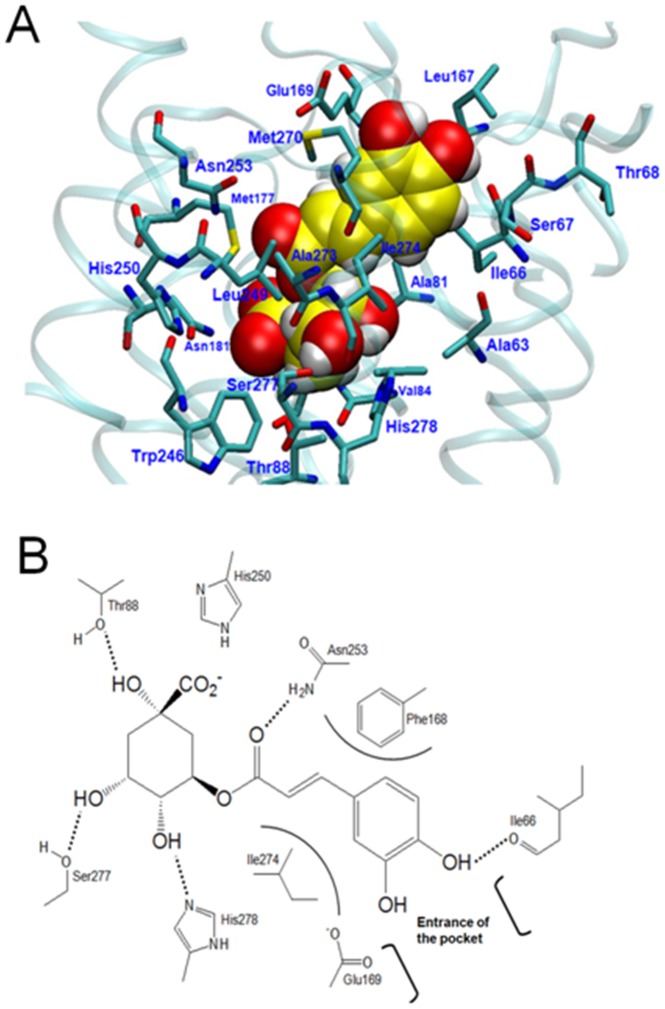
Predicted binding conformation of the chlorogenic acid inside A_2A_ receptor binding pocket. A) Alignment of docked structures of chlorogenic acid on X-ray reference structure of A_2A_ receptor binding pocket. B) Pocket of A_2A_ receptor occupied by chlorogenic acid.

Docking experiments showed that chlorogenic acid is well suited to the active site of the adenosine A_2A_ receptor (figure 12). The (2*E*)-3-(3,4-dihydroxyphenyl)-2-propenoyl group of the chlorogenic acid is located at the entrance of the binding pocket between the residues Ile274 at H7 and Phe168 at the extracellular loop 2 (EL2). The OH at position 4 of the 3,4-dihydroxyphenyl moiety forms a HB with the backbone carbonyl of Ile66 at H2, while the OH at position 3 is oriented towards Glu169 at EL2. The carbonyl oxygen of the propenoyl moiety forms a HB with the side-chain amine group of Asn253 at H6. The [(1*R*,2*R*,3*R*,5*S*)-5-carboxy-2,3,5-trihydroxycyclohexyl]oxy group is located deeper in the pocket of the adenosine A_2A_ receptor occupied by the ribose group of adenosine [32]. This is expected since this group contains a large number of hydroxyl groups like the ribose group. The OH groups at positions 2, 3, and 5 form HBs with the residues His278 at H7, Ser277 at H7, and Thr88 at H3, respectively. Meanwhile, the carboxylate group at position 5 is oriented towards the residue His250 at H6. These residues have been previously identified as relevant for the binding of A_2A_ ligands, such as adenosine and NECA [32].

## Discussion

We have demonstrated that chlorogenic acid possesses antiplatelet activity, reduces platelet release of atherosclerotic-related inflammatory mediators (sP-selectin, sCD40L, CCL5 and IL-1β) and inhibits *in vivo* thrombus formation. In addition, we have demonstrated for the first time that antiplatelet and antithrombotic effects shown by chlorogenic acid are associated byA_2A_ receptor activation and without significant bleeding.

Platelets play a key role in thrombosis and antiplatelet therapies, and may prevent as well as treat thrombotic diseases [33]. Aspirin is the most studied and widely used antiplatelet agent for stroke prevention [34]. The clopidogrel/aspirin combination provides increased efficacy compared with aspirin alone for prevention of vascular events. However, the increased risk of major bleeding offsets a portion of its benefit [35]. Therefore, in addition to the increasing incidence of and mortality from CVD, there is much room for further improvement of antiplatelet treatments and the development of novel antiplatelet agents with increased efficacy and safety profile.

Platelet activation plays an important role in physiological hemostasis and pathological thrombosis [36]. P-selectin may play a central role in platelet interactions not only with endothelial cells and leukocytes but also with other platelets. Since P-selectin determines the size and stability of platelet aggregates, it may be important in arterial thrombosis [31]. In this context, our results suggest that chlorogenic acid inhibition of platelet P-selectin expression may be involved in preventing collagen-induced platelet adhesion and aggregation under flow conditions. In addition, the slight inhibition of GPIIb/IIIa activation by chlorogenic acid can prevent platelet aggregation induced by activating factors.

In the last decade, several reports have supported that the secretion of platelet pro-inflammatory mediators (sP-selectin, sCD40L and CCL5, among others) play a pathogenic role in atherothrombosis [7], [8]. In this regard, this study demonstrates, for the first time that chlorogenic acid is also capable of diminishing platelet-derived inflammatory response through a lower release of sP-selectin, sCD40L, CCL5 and IL-1β.

Platelets are activated by different activators via complex signal pathways. Interestingly, chlorogenic acid inhibitory effects were detected upon the stimulation of a broad range of agonists (ADP, collagen, AA and TRAP-6). We also provide evidence that chlorogenic acid inhibition of platelet aggregation was in parallel with an increase of cAMP intraplatelet levels. The activation of human platelets is inhibited by intracellular cAMP- and cGMP-mediated pathways, and the importance of cAMP and cGMP in modulating platelet reactivity is well established [37]. Changes in platelet shape can be antagonized by PKA (cAMP-dependent) activation but not by PKG (cGMP-dependent) [38]. In this way, cAMP-induced inhibition of platelet P-selectin expression is largely mediated by PKA activation [39]. In fact, an increase of intraplatelet levels of cAMP has been shown to downregulate P2Y1R expression [40], maintain GPVI in a monomeric form, keep platelets in a resting state [41] and inhibited sCD40L release from platelets via the HSP27/p38 MAP kinase pathway [42]. Following this, the molecular mechanism by which chlorogenic acid increases cAMP formation was investigated.

In this study, chlorogenic acid was found to stimulate cAMP, inhibits platelet aggregation and platelet inflammatory mediators, and these effects were inhibited by SQ22536. Since the antiplatelet activity of chlorogenic acid is by adenylate cyclase/cAMP/PKA signaling pathway, we investigated whether chlorogenic acid activates the platelet A_2A_ receptor. Thus, ZM 241385 attenuated antiplatelet activity of chlorogenic acid, confirming that A_2A_ receptor activation could mediate the antiplatelet effects of chlorogenic acid. Considering that antiplatelet activity of chlorogenic acid was inhibited by both SQ22536 and ZM 241385, we explored chlorogenic acid potential antiplatelet effects via activation of A_2A_ receptor, the major subtype of adenosine receptors found in platelets [40]. Molecular modeling revealed that chlorogenic acid establishes an hydrogen bond and hydrophobic interactions with amino acids that typically participate in the interactions with A_2A_ ligands, such as adenosine and NECA [32], [43].

Taking into consideration all these findings, we provide further *in vivo* evidence of such *in vitro*-related chlorogenic acid- antithrombotic effects. In this study, by using a murine model of real time thrombus formation we demonstrated that chlorogenic acid administration diminished thrombus growth with a kinetics of thrombus inhibition similar to that of aspirin, a widely used antiplatelet agent. Moreover, all antiplatelet drugs currently available inevitably increase bleeding risk at the antithrombotic doses, which limits the achievement of improved antithrombotic effects by increasing doses [44]. This study reports that chlorogenic acid possesses antithrombotic efficacy without significant bleeding; this makes chlorogenic acid more promising as an antiplatelet agent for further development to prevent and treat atherothrombotic diseases.

In this study, *in vitro* platelets were treated with chlorogenic acid at mmol/L concentrations. However, in arterial thrombus formation *in vivo,* the administration of chlorogenic acid showed the same reductions in occlusion size when compared with adenosine (adenosine is present in the circulation at µmol/L concentrations) (data not shown). Therefore, in this article chlorogenic acid was studied in mmol/L concentrations to establish the mechanism of antiplatelet action. In addition, *in vivo* both chlorogenic acid and adenosine have the same bioactivity. Above might be explained due to one third of chlorogenic acid is absorbed in the small intestine in humans. This implies that part of chlorogenic acid from foods (apples, pears, berries, artichokes and aubergines, among others) will enter the blood circulation and thus can induce biological effects [45]. Chlorogenic acid inhibits LDL oxidation, reduces plasma glucose and blood pressure, and therefore might protect against CVD [46], [47], [48], [49].

In conclusion, we showed that chlorogenic acid inhibited platelet activation by the following mechanism of action: A_2A_ receptor/adenylate cyclase/cAMP/PKA activation, and consequently, suppression of activation of the GPIIb/IIIa receptor and platelet secretion. In addition, ongoing studies should be carried out on the subject of expanding chlorogenic acid properties in the setting of thrombosis and bleeding events.

## References

[1] World Health Organization (2004) WHO publishes definitive atlas on global heart disease and stroke epidemic (The atlas of heart disease and stroke). GENEVA. 15902773

[2] AHA Statistical Fact Sheet (2003) International Cardiovascular Disease Statistics. American Heart Association.

[3] Badimon L, Padro T, Vilahur G (2012) Atherosclerosis, platelets and thrombosis in acute ischaemic heart disease. Eur Heart J Acute Cardiovasc Care 1: 60–74. 24062891 10.1177/2048872612441582 PMC3760546

[4] da Costa Martins PA, van Gils JM, Mol A, Hordijk PL, Zwaginga JJ (2006) Platelet binding to monocytes increases the adhesive properties of monocytes by up-regulating the expression and functionality of beta1 and beta2 integrins. J Leukoc Biol 79: 499–507. 10.1189/jlb.0605318 16415171

[5] da Costa Martins P, van den Berk N, Ulfman LH, Koenderman L, Hordijk PL, et al (2004) Platelet-monocyte complexes support monocyte adhesion to endothelium by enhancing secondary tethering and cluster formation. Arterioscler Thromb Vasc Biol 24: 193–199. 14615387 10.1161/01.ATV.0000106320.40933.E5

[6] Massberg S, Schulz C, Gawaz M (2003) Role of platelets in the pathophysiology of acute coronary syndrome. Semin Vasc Med 3: 147–162. 15199478 10.1055/s-2003-40673

[7] Aukrust P, Muller F, Ueland T, Berget T, Aaser E, et al (1999) Enhanced levels of soluble and membrane-bound CD40 ligand in patients with unstable angina. Possible reflection of T lymphocyte and platelet involvement in the pathogenesis of acute coronary syndromes. Circulation 100: 614–620. 10441098 10.1161/01.cir.100.6.614

[8] Nurden AT (2011) Platelets, inflammation and tissue regeneration. Thromb Haemost 105 Suppl 1 S13–33. 21479340 10.1160/THS10-11-0720

[9] Barrett NE, Holbrook L, Jones S, Kaiser WJ, Moraes LA, et al (2008) Future innovations in anti-platelet therapies. Br J Pharmacol 154: 918–939. 18587441 10.1038/bjp.2008.151 PMC2451055

[10] Raju NC, Eikelboom JW (2012) The aspirin controversy in primary prevention. Curr Opin Cardiol 27: 499–507. 22874127 10.1097/HCO.0b013e328356ae95

[11] Plaza M, Cifuentes A, Ibañez E (2008) In the search of new functional food ingredients from algae. Trends in Food Science and Technology 19: 31–39.

[12] Wang Y, Wang J, Guo L, Gao X (2012) Antiplatelet effects of qishen yiqi dropping pill in platelets aggregation in hyperlipidemic rabbits. Evid Based Complement Alternat Med 2012: 1–5. 10.1155/2012/205451 PMC3434420 22969824

[13] Fuentes E, Forero-Doria O, Carrasco G, Marican A, Santos LS, et al (2013) Effect of tomato industrial processing on phenolic profile and antiplatelet activity. Molecules 18: 11526–11536. 24048285 10.3390/molecules180911526 PMC6270454

[14] Fuentes E, Palomo I (2013) Relationship between Platelet PPARs, cAMP Levels, and P-Selectin Expression: Antiplatelet Activity of Natural Products. Evid Based Complement Alternat Med 2013: 1–10. 10.1155/2013/861786 PMC3845334 24324520

[15] Karunanidhi A, Thomas R, van Belkum A, Neela V (2013) In vitro antibacterial and antibiofilm activities of chlorogenic acid against clinical isolates of Stenotrophomonas maltophilia including the trimethoprim/sulfamethoxazole resistant strain. Biomed Res Int 2013: 1–7. 10.1155/2013/392058 PMC3591175 23509719

[16] Meng S, Cao J, Feng Q, Peng J, Hu Y (2013) Roles of Chlorogenic Acid on Regulating Glucose and Lipids Metabolism: A Review. Evid Based Complement Alternat Med 2013: 1–11. 10.1155/2013/801457 PMC3766985 24062792

[17] Hwang SJ, Kim YW, Park Y, Lee HJ, Kim KW (2013) Anti-inflammatory effects of chlorogenic acid in lipopolysaccharide-stimulated RAW 264.7 cells. Inflamm Res 2013: 1–10. 10.1007/s00011-013-0674-4 24127072

[18] Amin RP, Kunaparaju N, Kumar S, Taldone T, Barletta MA, et al (2013) Structure elucidation and inhibitory effects on human platelet aggregation of chlorogenic acid from Wrightia tinctoria. J Complement Integr Med 10: 1–8. 10.1515/jcim-2012-0048 23735478

[19] Bijak M, Saluk J, Ponczek MB, Nowak P (2013) Antithrombin effect of polyphenol-rich extracts from black chokeberry and grape seeds. Phytother Res 27: 71–76. 22473647 10.1002/ptr.4682

[20] Frojmovic M, Wong T, van de Ven T (1991) Dynamic measurements of the platelet membrane glycoprotein IIb-IIIa receptor for fibrinogen by flow cytometry. I. Methodology, theory and results for two distinct activators. Biophys J 59: 815–827. 1905966 10.1016/S0006-3495(91)82294-9 PMC1281247

[21] Conant CG, Schwartz MA, Nevill T, Ionescu-Zanetti C (2009) Platelet adhesion and aggregation under flow using microfluidic flow cells. J Vis Exp 32: 1–3. 10.3791/1644 PMC3164058 19859055

[22] Appeldoorn CC, Bonnefoy A, Lutters BC, Daenens K, van Berkel TJ, et al (2005) Gallic acid antagonizes P-selectin-mediated platelet-leukocyte interactions: implications for the French paradox. Circulation 111: 106–112. 15630039 10.1161/01.CIR.0000151307.10576.02

[23] Marks DC, Belov L, Davey MW, Davey RA, Kidman AD (1992) The MTT cell viability assay for cytotoxicity testing in multidrug-resistant human leukemic cells. Leuk Res 16: 1165–1173. 1361210 10.1016/0145-2126(92)90114-m

[24] Przyklenk K, Whittaker P (2007) Adaptation of a photochemical method to initiate recurrent platelet-mediated thrombosis in small animals. Lasers Med Sci 22: 42–45. 17333457 10.1007/s10103-006-0410-1

[25] De Clerck F, Goossens J, Reneman R (1976) Effects of anti-inflammatory, anticoagulant and vasoactive compounds on tail bleeding time, whole blood coagulation time and platelet retention by glass beads in rats. Thromb Res 8: 179–193. 10.1016/0049-3848(76)90261-9 1251349

[26] Friesner R, Banks J, Murphy R, Halgren T, Klicic J, et al (2004) Glide: a new approach for rapid, accurate docking and scoring. 1. Method and assessment of docking accuracy. Journal of Medicinal Chemistry 47: 1739–1749. 15027865 10.1021/jm0306430

[27] Munoz C, Adasme F, Alzate-Morales JH, Vergara-Jaque A, Kniess T, et al (2012) Study of differences in the VEGFR2 inhibitory activities between semaxanib and SU5205 using 3D-QSAR, docking, and molecular dynamics simulations. J Mol Graph Model 32: 39–48. 22070999 10.1016/j.jmgm.2011.10.005

[28] Onate B, Vilahur G, Ferrer-Lorente R, Ybarra J, Diez-Caballero A, et al (2012) The subcutaneous adipose tissue reservoir of functionally active stem cells is reduced in obese patients. FASEB J 26: 4327–4336. 22772162 10.1096/fj.12-207217

[29] Jorgensen W, Maxwell D, Tirado-Rives J (1996) Development and testing of the OPLS all-atom force field on conformational energetics and properties of organic liquids. Journal of the American Chemical Society 118: 11225–11236.

[30] Eldridge MD, Murray CW, Auton TR, Paolini GV, Mee RP (1997) Empirical scoring functions: I. The development of a fast empirical scoring function to estimate the binding affinity of ligands in receptor complexes. J Comput Aided Mol Des 11: 425–445. 9385547 10.1023/a:1007996124545

[31] Merten M, Thiagarajan P (2000) P-selectin expression on platelets determines size and stability of platelet aggregates. Circulation 102: 1931–1936. 11034941 10.1161/01.cir.102.16.1931

[32] Lebon G, Warne T, Edwards PC, Bennett K, Langmead CJ, et al (2011) Agonist-bound adenosine A2A receptor structures reveal common features of GPCR activation. Nature 474: 521–525. 21593763 10.1038/nature10136 PMC3146096

[33] Ji X, Hou M (2011) Novel agents for anti-platelet therapy. J Hematol Oncol 4: 1–7. 22053759 10.1186/1756-8722-4-44 PMC3224753

[34] Albers GW, Amarenco P, Easton JD, Sacco RL, Teal P (2001) Antithrombotic and thrombolytic therapy for ischemic stroke. Chest 119: 300S–320S. 11157656 10.1378/chest.119.1_suppl.300s

[35] Albers GW, Amarenco P (2001) Combination therapy with clopidogrel and aspirin: can the CURE results be extrapolated to cerebrovascula patients? Stroke 32: 2948–2949. 11740003 10.1161/hs1201.100829

[36] Offermanns S (2006) Activation of platelet function through G protein-coupled receptors. Circ Res 99: 1293–1304. 17158345 10.1161/01.RES.0000251742.71301.16

[37] Walter U, Eigenthaler M, Geiger J, Reinhard M (1993) Role of cyclic nucleotide-dependent protein kinases and their common substrate VASP in the regulation of human platelets. Adv Exp Med Biol 344: 237–249. 8209791 10.1007/978-1-4615-2994-1_19

[38] Jensen BO, Selheim F, Doskeland SO, Gear AR, Holmsen H (2004) Protein kinase A mediates inhibition of the thrombin-induced platelet shape change by nitric oxide. Blood 104: 2775–2782. 15265792 10.1182/blood-2004-03-1058

[39] Libersan D, Rousseau G, Merhi Y (2003) Differential regulation of P-selectin expression by protein kinase A and protein kinase G in thrombin-stimulated human platelets. Thromb Haemost 89: 310–317. 12574812

[40] Yang D, Chen H, Koupenova M, Carroll SH, Eliades A, et al (2010) A new role for the A2b adenosine receptor in regulating platelet function. J Thromb Haemost 8: 817–827. 20102488 10.1111/j.1538-7836.2010.03769.x

[41] Loyau S, Dumont B, Ollivier V, Boulaftali Y, Feldman L, et al (2012) Platelet glycoprotein VI dimerization, an active process inducing receptor competence, is an indicator of platelet reactivity. Arterioscler Thromb Vasc Biol 32: 778–785. 22155453 10.1161/ATVBAHA.111.241067

[42] Enomoto Y, Adachi S, Doi T, Natsume H, Kato K, et al (2011) cAMP regulates ADP-induced HSP27 phosphorylation in human platelets. Int J Mol Med 27: 695–700. 21373747 10.3892/ijmm.2011.637

[43] Jaakola VP, Griffith MT, Hanson MA, Cherezov V, Chien EY, et al (2008) The 2.6 angstrom crystal structure of a human A2A adenosine receptor bound to an antagonist. Science 322: 1211–1217. 18832607 10.1126/science.1164772 PMC2586971

[44] Serebruany VL, Malinin AI, Eisert RM, Sane DC (2004) Risk of bleeding complications with antiplatelet agents: meta-analysis of 338,191 patients enrolled in 50 randomized controlled trials. Am J Hematol 75: 40–47. 14695631 10.1002/ajh.10451

[45] Olthof MR, Hollman PC, Zock PL, Katan MB (2001) Consumption of high doses of chlorogenic acid, present in coffee, or of black tea increases plasma total homocysteine concentrations in humans. Am J Clin Nutr 73: 532–538. 11237928 10.1093/ajcn/73.3.532

[46] Laranjinha JA, Almeida LM, Madeira VM (1994) Reactivity of dietary phenolic acids with peroxyl radicals: antioxidant activity upon low density lipoprotein peroxidation. Biochem Pharmacol 48: 487–494. 8068036 10.1016/0006-2952(94)90278-x

[47] Nardini M, D'Aquino M, Tomassi G, Gentili V, Di Felice M, et al (1995) Inhibition of human low-density lipoprotein oxidation by caffeic acid and other hydroxycinnamic acid derivatives. Free Radic Biol Med 19: 541–552. 8529913 10.1016/0891-5849(95)00052-y

[48] Watanabe T, Arai Y, Mitsui Y, Kusaura T, Okawa W, et al (2006) The blood pressure-lowering effect and safety of chlorogenic acid from green coffee bean extract in essential hypertension. Clin Exp Hypertens 28: 439–449. 16820341 10.1080/10641960600798655

[49] Bassoli BK, Cassolla P, Borba-Murad GR, Constantin J, Salgueiro-Pagadigorria CL, et al (2008) Chlorogenic acid reduces the plasma glucose peak in the oral glucose tolerance test: effects on hepatic glucose release and glycaemia. Cell Biochem Funct 26: 320–328. 17990295 10.1002/cbf.1444

